# A phase 2a randomized clinical trial of intravenous vedolizumab for the treatment of steroid-refractory intestinal acute graft-versus-host disease

**DOI:** 10.1038/s41409-021-01356-0

**Published:** 2021-06-09

**Authors:** Yngvar Fløisand, Mark A. Schroeder, Patrice Chevallier, Dominik Selleslag, Steven Devine, Anne S. Renteria, Mohamad Mohty, Ibrahim Yakoub-Agha, Chunlin Chen, Andrejus Parfionovas, Syed Quadri, Johan Jansson, Mona Akbari, Yi-Bin Chen

**Affiliations:** 1 Department of Haematology, Oslo University Hospital, Rikshospitalet, Oslo, Norway; 2 Centre for Cancer Cell Reprogramming, Department of Molecular Cell Biology, Oslo University Hospital, Oslo, Norway; 3 Division of Oncology, Washington University School of Medicine, St. Louis, MO USA; 4 CHU de l’Hôtel-Dieu, service d’hématologie clinique, place A. Ricordeau, Nantes, France; 5 AZ Sint-Jan Brugge-Oostende, Brugge, Belgium; 6 Center for International Blood and Marrow Transplant Research (CIMBTR) Minneapolis Campus, Minneapolis, MN USA; 7 Hematology Oncology, Cellular and Gene Therapy, ICON Clinical Research Service, New York, NY USA; 8 Hematology Department, AP-HP, Hôpital Saint-Antoine, Sorbonne Université, and INSERM UMRs 938, Paris, France; 9 CHU de Lille, Univ Lille, INSERM U1286, Infinite, Lille, France; 10 Millennium Pharmaceuticals Inc, Cambridge, MA USA; 11 Hematopoietic Cell Transplant and Cellular Therapy Program, Massachusetts General Hospital, Boston, MA USA

**Keywords:** Graft-versus-host disease, Drug development, Molecularly targeted therapy

## Abstract

Steroid-refractory (SR) acute graft-versus-host disease (aGvHD) remains a significant complication after allogeneic hematopoietic cell transplantation. Systemic corticosteroids are first-line therapy for aGvHD, but apart from ruxolitinib, there are no approved treatments for SR aGvHD. Vedolizumab is approved for treatment of ulcerative colitis and Crohn’s disease, and may be effective for treatment of SR intestinal aGvHD. We conducted a phase 2a trial (NCT02993783) to evaluate the clinical efficacy, tolerability, and safety of vedolizumab 300 and 600 mg for SR intestinal aGvHD. This study was terminated before full enrollment was completed because early results failed to demonstrate positive proof-of-concept in efficacy. Before termination, 17 participants had enrolled and an early response in intestinal aGvHD was observed in 11 and eight participants at days 15 and 28, respectively. All adverse events observed were consistent with those expected in a population with SR intestinal aGvHD. Overall, vedolizumab did not meet the primary efficacy endpoint (overall response at day 28), likely owing to premature study drug discontinuation, lack of efficacy, and the competing risks inherent with a population with advanced SR intestinal aGvHD. Nevertheless, this study provides valuable insights into the considerations needed when conducting studies in patients with SR intestinal aGvHD.

## Introduction

Allogeneic hematopoietic cell transplantation is an important and potentially curative therapy for many hematologic malignancies, but carries the significant risk of graft-versus-host disease (GvHD) [1, 2]. Acute GvHD (aGvHD), which generally occurs in the first few months after allogeneic hematopoietic stem cell transplantation, typically involves the skin, liver, and/or intestine [2–4].

After conventional allogeneic hematopoietic cell transplantation, the incidence of aGvHD ranges from 10% to 80% (dependent on certain clinical risk factors) in patients receiving T-cell-replete allogeneic grafts, despite standard measures of prophylaxis [5–7].

First-line therapy for patients who develop aGvHD is systemic corticosteroids, but these are effective only in ~50% of patients, with durable responses observed in only one-third [8, 9]. Apart from ruxolitinib, which is associated with cytopenias, there are no other approved treatments for steroid-refractory (SR) aGvHD, which is associated with historical 2-year survival rates of <20% [10–12]. Furthermore, there are no targeted treatments for intestinal aGvHD, for the majority of morbidity and mortality associated with SR aGvHD [13].

Vedolizumab, a humanized immunoglobulin G1 monoclonal antibody directed against the α4β7 integrin expressed on lymphocytes and implicated in gastrointestinal (GI) trafficking, was developed for the treatment of moderate–severe ulcerative colitis and Crohn’s disease [14–16]. Vedolizumab inhibits the interaction between α4β7 integrin and the mucosal addressin cell adhesion molecule-1 (MAdCAM-1), preventing the migration of gut-homing leukocytes into the GI mucosa [17–20]. Preclinical studies have suggested that blocking this interaction may prevent intestinal aGvHD, and multiple series have suggested that vedolizumab may have efficacy for the treatment of SR intestinal aGvHD [21–23].

Here we present data from a phase 2a clinical trial (NCT02993783), which prospectively investigated the clinical efficacy, tolerability, and safety of vedolizumab for the treatment of SR intestinal aGvHD.

## Methods

### Study design

This was a phase 2a, randomized, parallel, open-label study with two dose groups evaluating the clinical efficacy, tolerability, and safety of vedolizumab (Hospira McPherson, KS, USA) IV for treatment of SR intestinal aGvHD.

### Recruitment and consent

Participants were recruited and enrolled from the investigator’s practice or on referrals from other physicians. If alternative recruitment strategies were employed (such as advertisement) these were reviewed by the institutional review board (IRB)/independent ethics committee (IEC). All participants provided written informed consent before any study-required procedures were conducted, unless such procedure formed part of their standard of care.

### Eligibility criteria

Participants were 18 years or older, had undergone a first allogeneic hematopoietic cell transplant, and had developed SR intestinal aGvHD (worsening or no improvement after 5–7 days of methylprednisolone 2 mg/kg/day or equivalent, or lack of a complete response after 14 days of methylprednisolone 2 mg/kg/day or equivalent (Supplementary Table 1)). Participants who developed intestinal aGvHD while receiving systemic therapy for other aGvHD organ manifestations were also eligible after 5–7 days, even if intestinal aGvHD had not been present for the entire duration. Participants were required to have Grade B–D aGvHD with intestinal involvement and an Eastern Cooperative Oncology Group (ECOG) Performance Status of 0–3. Patients who had received any systemic agents beyond corticosteroids for the treatment of aGvHD were excluded. Detailed inclusion and exclusion criteria are listed in Supplementary Table 2.

### Treatments

The intravenous (IV) doses and schedule of vedolizumab were selected based on those approved for the treatment of ulcerative colitis and Crohn’s disease (300 mg IV every 8 weeks) [14]. Because SR intestinal aGvHD may require higher doses of vedolizumab to achieve target saturation, owing to hypoalbuminemia, a dose of vedolizumab 600 mg IV was also evaluated [14, 16, 24, 25].

Participants were randomized 1:1 to receive 300 or 600 mg doses of vedolizumab IV on days 1, 15, 43, 71, and 99 on the basis of evidence from a previous case series [26]. Investigators used an interactive voice or web response technology system at screening to obtain the participant study number and randomize them to receive 300 or 600 mg doses of vedolizumab. Participants who responded to, and tolerated all five planned doses of vedolizumab, and who developed recurrent symptoms of intestinal aGvHD following discontinuation of therapy were permitted to receive additional doses of vedolizumab for up to 1 year from the first dose of the study drug (extension phase). Other medications considered necessary for the safety and wellbeing of the participant were permitted at the discretion of the investigator.

### Sample size

It was intended that after ten participants had enrolled in each dose group and had been followed through day 28, safety, tolerability, efficacy, and pharmacokinetic (PK) results would be assessed to determine an appropriate dose for subsequent participants. The group at the chosen dose would have been expanded by approximately 18 additional participants to assess the tolerability and efficacy of vedolizumab further (approximately 38 patients in total were planned for enrollment). However, early results failed to demonstrate a positive proof-of-concept in efficacy (described below) and the trial was terminated early.

### Study endpoints

The primary endpoints of the study were overall response (OR) (defined as either a partial response, a very good partial response, or a complete response—Supplementary Table 1) at day 28 and incidence of treatment-emergent serious adverse events (TEAEs) following the administration of the first dose of vedolizumab to day 28.

Secondary endpoints included overall survival at 6 and 12 months, complete response at day 28, intestinal OR at day 28, and TEAEs and/or serious adverse events following the first dose of vedolizumab through 18 weeks after the last dose of vedolizumab (treatment exposure period). PK characterization of vedolizumab and the presence of anti-vedolizumab antibodies at baseline and at the end of the exposure period were also performed, and durable intestinal OR rate (intestinal OR achieved at day 28 and maintained OR at day 43) was evaluated as an exploratory endpoint.

All participants were planned to be followed for overall survival every 3 months until death, withdrawal of consent, termination of study by the sponsor, or for a maximum of 1 year after the last subject was enrolled in the study.

### Measurements

Severity of aGvHD was assessed at each visit using the Blood and Marrow Transplant Clinical-Trials Network (BMT CTN)-modified International Blood and Marrow Transplant Research (IBMTR) database index (Supplementary Table 3) [27, 28]. The severity (Stage 0–4) of skin, liver, and intestinal GvHD was based on aGvHD clinical-stage criteria.

Blood samples for PK analysis of vedolizumab were collected as per the schedule outlined in Supplementary Table 4. Vedolizumab serum concentrations were measured by enzyme-linked immunosorbent assay with a validated range of 0.20–8.00 μg/mL. Immunogenicity was evaluated using blood sampling for anti-vedolizumab antibodies in pre-dose serum on day 1, and on days 22, 71, and 99.

### Statistics

A Bayesian statistical approach was planned for efficacy evaluation. An interim analysis was planned when all participants had reached day 28 (10 participants in each group). A positive proof-of-concept in efficacy was considered if the posterior probability of day 28 OR rate >54% was at least 85% and day 28 OR rate being at least 70% was at least 75%. Early review of patient-level data was performed ahead of the planned analysis to assess safety, PK, and efficacy data in order to facilitate decision-making in the study.

Enrolled participants who received any dose of study drug comprised the safety analysis set. The efficacy analysis set included all participants from the safety analysis set who had a baseline efficacy assessment and at least one postbaseline efficacy assessment. The PK analysis set comprised all participants in the safety analysis set who had at least one post-dose PK sample collected.

Approximately 38 patients were planned for enrollment. The sample size was based on clinical considerations and considered sufficient to determine a recommended dose and regimen and to describe the efficacy, tolerability, and safety of vedolizumab IV.

## Results

### Study population

On the basis of an early review of patient-level data and the totality of evidence available, it was concluded that the ability to evaluate overall efficacy was impaired owing to (a) the competing risks in the population, (b) premature discontinuation of study drug, and (c) observed mortality. It was determined that the proportion of participants achieving the primary efficacy outcome was not expected to reach 54% and the study was terminated early (9 May 2018) by the sponsor. Participants were recruited and followed from 28 April 2017 until study termination.

**Fig. 1:**
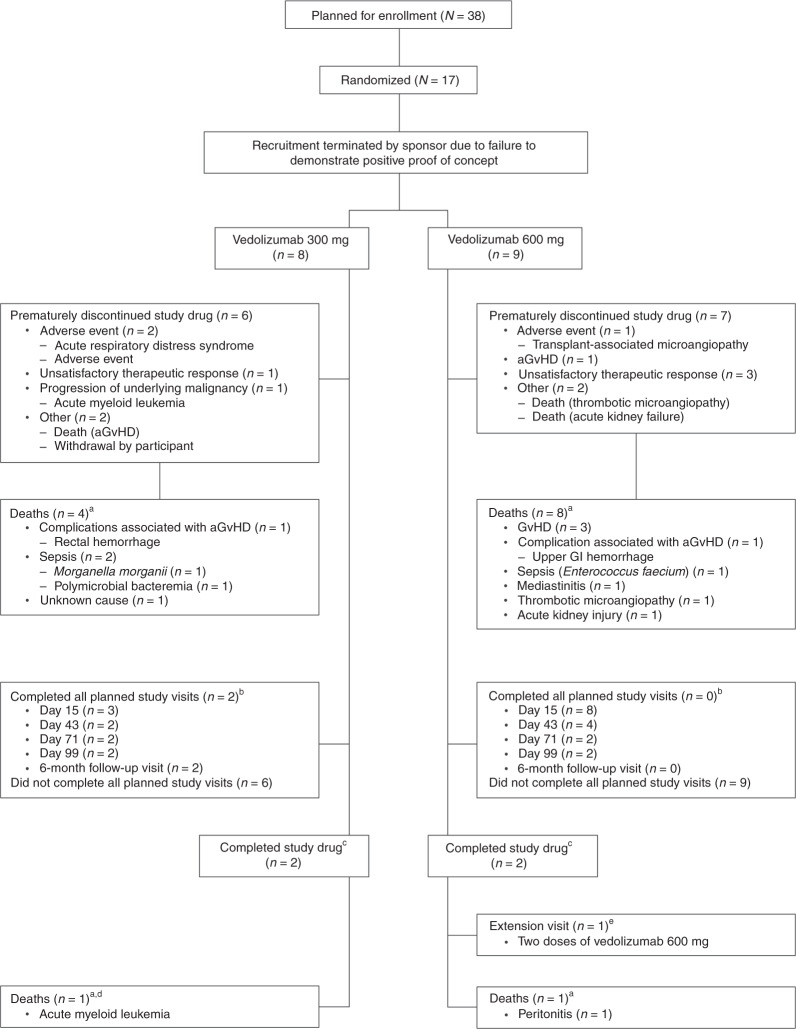
Flow diagram of participants. Data presented are from the safety analysis set. ^a^No deaths were considered related to study drug by the investigator. ^b^Completed all planned study visits includes participants who completed all protocol required assessments and did not discontinue the study early. ^c^Completed study drug includes participants who completed all five planned doses. ^d^An additional participant in the vedolizumab IV 300 mg group died after the end of the treatment-emergent period (i.e., 18 weeks after the last dose of study drug). ^e^Upon review and agreement by the medical monitor, participants who responded to and tolerated all five planned doses of vedolizumab and who developed recurrent symptoms of intestinal aGvHD following discontinuation of therapy (i.e., after the fifth dose) could enter an extension phase to receive vedolizumab 300 mg every 2 weeks for two doses followed by every 4 weeks for up to 1 year from the first dose of study drug. A dose other than 300 mg and/or a frequency of administration other than every 4 weeks may have been chosen based on accumulating safety, tolerability, efficacy, and pharmacokinetic results. *aGvHD* acute graft-versus-host disease, *IV* intravenous.

Before termination, 17 participants were enrolled and treated at 11 sites in Belgium, France, Norway, and the USA (Fig. 1). Of these, 13 participants completed day 28, four participants (23.5%) completed all five planned doses of vedolizumab (two from each dose group), and one participant entered the extension phase and received two additional doses of vedolizumab 600 mg. The median number of doses in each treatment group was 2.

### Demographics and disease characteristics

Baseline characteristics are presented in Table 1. Overall median age was 57 years (range, 34–74 years), with four participants (23.5%) aged 65 years or older. Five participants (29.4%) had a baseline ECOG Performance Status of 3.

**Table 1:** Baseline characteristics.

	Number of participants *n*, (%)		
	Vedolizumab 300 mg (*n* = 8)	Vedolizumab 600 mg (*n* = 9)	Total (*N* = 17)
Age (years)^a^			
Mean (SD)	54.1 (10.45)	59.0 (10.87)	56.7 (10.64)
Minimum, maximum	40, 74	34, 70	34, 74
Sex, *n* (%)			
Male	2 (25.0)	5 (55.6)	7 (41.2)
Female	6 (75.0)	4 (44.4)	10 (58.8)
ECOG performance status *n*, (%)^b^			
0	2 (25.0)	0	2 (11.8)
1	3 (37.5)	2 (22.2)	5 (29.4)
2	1 (12.5)	4 (44.4)	5 (29.4)
3	2 (25.0)	3 (33.3)	5 (29.4)
4	0	0	0
aGvHD characteristics, grade^c^			
Grade A	0	0	0
Grade B	2 (25.0)	3 (33.3)	5 (29.4)
Grade C	4 (50.0)	4 (44.4)	8 (47.1)
Grade D	2 (25.0)	2 (22.2)	4 (23.5)
aGvHD characteristics, involvements			
Intestinal only	7 (87.5)	5 (55.6)	12 (70.6)
Skin and intestinal	1 (12.5)	3 (33.3)	4 (23.5)
Liver and intestinal	0	1 (11.1)	1 (5.9)
Skin, liver, and intestinal	0	0	0
aGvHD characteristics, GI tract^d^			
Stage 0	0	0	0
Stage 1	1 (12.5)	3 (33.3)	4 (23.5)
Stage 2	1 (12.5)	0	1 (5.9)
Stage 3	4 (50.0)	4 (44.4)	8 (47.1)
Stage 4	2 (25.0)	2 (22.2)	4 (23.5)
Conditioning regimen			
Myeloablative transplant	4 (50.0)	3 (33.3)	7 (41.2)
Non-myeloablative or reduced-intensity transplant	4 (50.0)	6 (66.7)	10 (58.8)
Stem cell source			
Bone marrow	1 (12.5)	1 (11.1)	2 (11.8)
Peripheral blood	7 (87.5)	8 (88.9)	15 (88.2)
HLA compatibility			
Matched	6 (75.0)	6 (66.7)	12 (70.6)
Mismatched	1 (12.5)	1 (11.1)	2 (11.8)
Haploidentical	1 (12.5)	2 (22.2)	3 (17.6)
CMV IgG antibody mismatch, donor/recipient			
Positive/positive	2 (25.0)	1 (11.1)	3 (17.6)
Positive/negative	2 (25.0)	2 (22.2)	4 (23.5)
Negative/positive	2 (25.0)	2 (22.2)	4 (23.5)
Negative/negative	2 (25.0)	4 (44.4)	6 (35.3)

*CMV* cytomegalovirus, *ECOG* Eastern Cooperative Oncology Group, *IgG* immunoglobulin G, *GI* gastrointestinal, *GvHD* graft-versus-host disease, *HLA* human leukocyte antigen, *SD* standard deviation.

^a^Age at the date of informed consent.

^b^(0) Fully active, able to carry on all predisease performance without restriction, (1) restricted in physically strenuous activity, but ambulatory and able to carry out work of a light or sedentary nature (e.g., light housework, office work), (2) ambulatory and capable of all self-care, but unable to carry out any work activities, up and about more than 50% of waking hours, (3) capable of only limited self-care, confined to bed or chair more than 50% of waking hours, (4) completely disabled, cannot carry on any self-care, totally confined to bed or chair.

^c^Grades were derived using the Blood and Marrow Transplant Clinical Trials Network (BMT CTN)-modified International Blood and Marrow Transplant Research (IBMTR) database index.

^d^Stage is based on aGvHD clinical stage criteria (Supplementary Table 3).

A total of 12 participants (70.6%) had isolated intestinal aGvHD, four (23.5%) had skin and intestinal involvement, and one (5.9%) had liver and intestinal involvement. Twelve participants (70.6%) had Grade C or D aGvHD (eight Grade C aGvHD, four Grade D aGvHD). At baseline, 13 participants (76.5%) had Stage 2 or greater intestinal aGvHD (one Stage 2, eight Stage 3, four Stage 4).

### Individual participant data

**Fig. 2:**
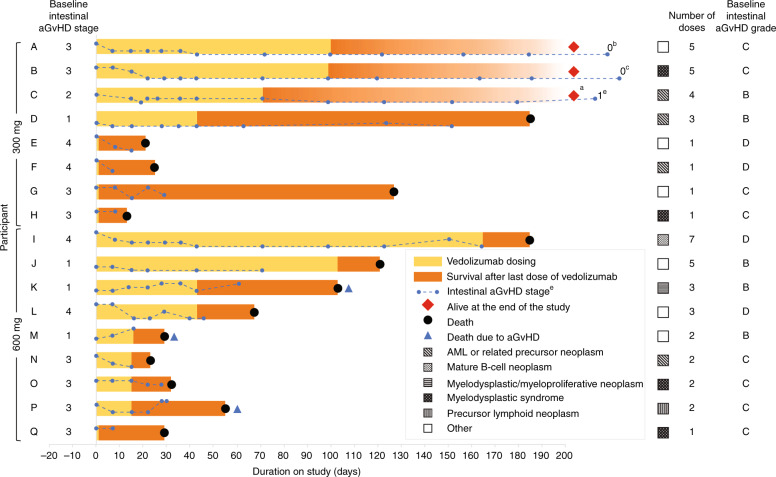
Duration of vedolizumab treatment, survival following final vedolizumab dose, and participant characteristics. Data presented are from the efficacy analysis set. ^a^Intestinal aGvHD stages (based on aGvHD clinical stage criteria) are indicated by blue circles and dashed trace (uppermost circle aGvHD stage 4; lowermost circle aGvHD stage 0) positioned on the day that the aGvHD assessment was performed. *Stage 0* no intestinal tract involvement, *Stage 1* > 500 mL diarrhea/day, *Stage 2* > 1000 mL diarrhea/day, *Stage 3* > 1500 mL diarrhea/day, *Stage 4* severe abdominal pain with or without ileus. ^b^day 255. ^c^day 276. ^d^Participant C was alive at the end of the study and on day 239 experienced recurrence of intestinal aGvHD at Stage 1. ^e^day 239. *aGvHD* acute graft-versus-host disease, *AML* acute myeloid leukemia, *GvHD* graft-versus-host disease.

Baseline disease characteristics and outcomes for each participant are presented in Supplementary Table 5A–C. A summary of the main participant characteristics and outcomes are outlined in Fig. 2.

### Discontinuation of study drug

Thirteen participants (76.5%) discontinued the study drug prematurely (Fig. 1). Five of these 13 participants (38.5%) discontinued due to progression of aGvHD (*n* = 1) or because of an unsatisfactory therapeutic response (*n* = 4). Four participants (30.8%) discontinued owing to ‘other’ reasons, including death (*n* = 3) and withdrawal (*n* = 1), three participants (23.1%) owing to a TEAE (thrombotic microangiopathy, acute respiratory distress syndrome, and adverse event, none of which were vedolizumab-related), and one participant (7.7%) owing to progression of an underlying malignancy (relapse of acute myeloid leukemia).

Of those who discontinued for reasons other than unsatisfactory therapeutic response or progression of aGvHD (*n* = 8), at the last study assessment only two were recorded as having no intestinal aGvHD involvement, suggesting that most had not responded to treatment at the time that they discontinued the study drug.

### Overall response

**Fig. 3:**
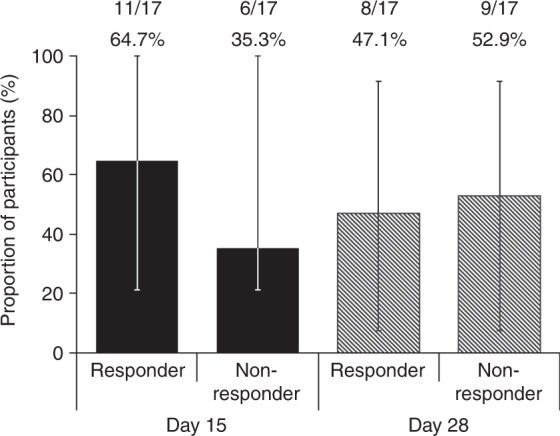
Proportion of participants with intestinal overall response at day 15 and day 18. Error bars represent 95% confidence interval of percentage of responders. Participants with missing data for endpoint determination were categorized as non-responders.

At day 28, a total of six participants (35.3%) had an OR in all organs involved, and six of the eight participants with an OR in intestinal aGvHD at day 28 experienced a durable response (response maintained through day 43). An OR in intestinal aGvHD was recorded in 11 participants (64.7%) and eight participants (47.1%) at day 15 and day 28, respectively (Fig. 3). A complete response in all organs involved was reported in two participants at day 15, in one at day 28, and in four at day 43.

### Overall survival

**Fig. 4:**
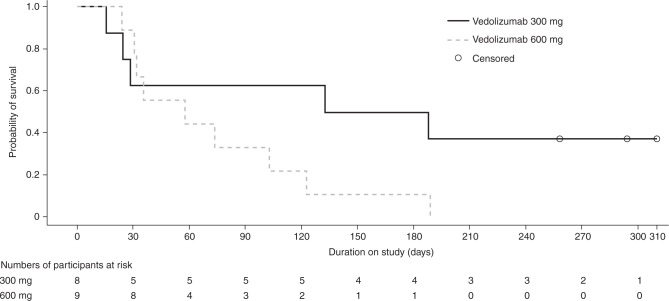
Kaplan–Meier estimates of overall survival. Data presented are from the efficacy analysis set. Overall survival was defined as the time from the date of enrollment to the date of death. Participants without documentation of death at the time of analysis were censored at the date last known to be alive.

At 6 months, five of the 17 participants (29.4%) were alive: four (50%; 4 of 8) in the vedolizumab 300 mg and one (11.1%; 1 of 9) in the vedolizumab 600 mg group, with a median overall survival of 161 days (range, 16–310 days) in the 300 mg group and 58 days (range, 24–189 days) in the 600 mg group (Fig. 4). At the time of study termination, three participants were alive, all from the 300 mg group. Owing to the early termination of the study, overall survival at 12 months was not determined.

### Pharmacokinetics

**Fig. 5:**
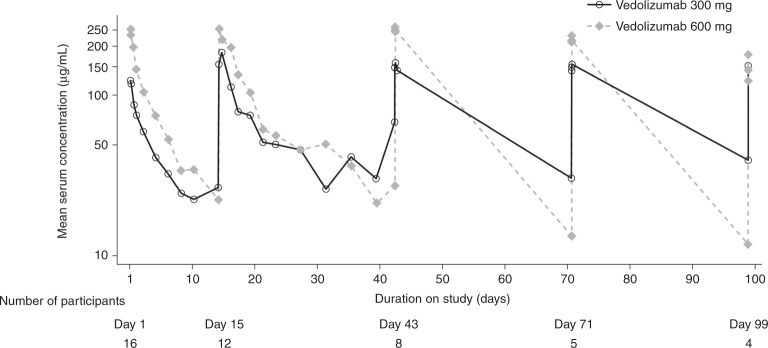
Mean serum vedolizumab concentration–time profiles following multiple IV infusions of vedolizumab 300 or 600 mg delivered over 30 min (semi-log scale). Data presented are from the pharmacokinetics analysis set (*n* = 16, eight participants from 300 and 600 mg dosing groups; participant N was excluded from the PK analysis set). The lower limit of quantification is 0.2 μg/mL. The concentrations below the limit of quantification are included as 0. Anomalous concentrations are excluded.

In general, exposure to vedolizumab was higher over time in participants receiving vedolizumab 600 mg compared with those receiving vedolizumab 300 mg and was comparable with that observed in previous multiple-dose PK studies in participants with ulcerative colitis and Crohn’s disease [31, 32]. Serum vedolizumab *C*_max_ was similar on all dosing days, whereas serum vedolizumab *C*_trough_ was variable, with no clear trends over time or between groups (Fig. 5). *C*_trough_ levels were lower in the 600 mg group versus the 300 mg group, but average *C*_trough_ concentrations still met the target concentration level of 10 µg/mL for participants with inflammatory bowel diseases [32, 33].

### Safety, tolerability, and treatment-emergent adverse events

An overview of TEAEs is presented in Supplementary Table 6. All participants in the study experienced at least one TEAE. A total of 50 serious adverse events were experienced by 15 participants (88.2%), most of which (*n* = 46 [92.0%]) were considered unrelated to the study drug. Overall, five participants (29.4%) experienced a TEAE, and two participants (11.8%) experienced a serious adverse event considered to be related to the study drug.

The most frequently reported adverse events were infections occurring in 15 participants (88.2%), including three participants (17.6%) with sepsis and four participants (23.5%) with cytomegalovirus (CMV) related infections, of which two (11.8%) were CMV colitis. The events of CMV colitis were both serious and considered related to the study drug for one participant (vedolizumab 600 mg). Commonly occurring infection-related TEAEs are presented in Supplementary Table 7.

No participants had detectable anti-vedolizumab antibodies at any scheduled time point.

### Deaths

During the TEAE reporting period, 13 participants (76.5%) died (Fig. 1). An additional participant died after the reporting period for TEAEs (18 weeks after the last dose of study drug). No deaths were considered by the investigator to be related to the study drug. The most common causes of death were attributed to complications associated progression of aGvHD (*n* = 5) or sepsis (*n* = 3). Other causes of death in the remaining participants (*n* = 5) who died during the TEAE period were due to the development of peritonitis (percutaneous endoscopic gastrostomy probe placement related), thrombotic microangiopathy, mediastinitis, or acute kidney injury; one cause of death was not reported. The death of the participant after the TEAE period was due to a fatal relapse of acute myeloid leukemia. Additional details at the time of death for each participant are provided in Table 2.

**Table 2:** Details of participant deaths during and after the TEAE reporting period.

Participant	Vedolizumab dose (mg)	MedDRA PT	As classified in text	Medically significant observations at time of death	Duration on study (days)
Death during the TEAE reporting period					
E	300	Bacterial sepsis	Sepsis	*Morganella morganii* infection	21
F	300	Sepsis	Sepsis	Polymicrobial bacteremia	25
G	300	Death^a^	Unknown	None reported	127
H	300	Rectal hemorrhage	aGvHD^b^	Rectal hemorrhage with CMV reactivation	13
I	600	Peritonitis	Peritonitis	Peritonitis following percutaneous endoscopic gastrostomy probe placement	185
J	600	Mediastinitis	Mediastinitis	Sepsis linked to *Citrobacter freundii*	121
K	600	Graft-versus-host disease	aGvHD^b^	Tremors, facial swelling, hypotension, and diarrhea	103
L	600	Thrombotic microangiopathy	Thrombotic microangiopathy	Stroke	70
M	600	Graft-versus-host disease	aGvHD^b^	Progressive aGvHD	29
N	600	Acute kidney injury	Acute kidney injury	Sepsis (bacterial infection not recorded), lower GI hemorrhage (progression of lower GI bleed)	23
O	600	Enterococcal sepsis	Sepsis	*Enterococcus faecium* infection, aGvHD liver, progression of aGvHD	32
P	600	Graft-versus-host disease in gastrointestinal tract	aGvHD^b^	Worsening of GI aGvHD	55
Q	600	Upper gastrointestinal hemorrhage	aGvHD^b^	Upper gastrointestinal hemorrhage and disseminated intravascular coagulation	29
Death after study termination					
D	300	Acute myeloid leukemia^c^	Acute myeloid leukemia	None reported	185
Alive at the study end					
A	300^d^	–		–	N/A
B	300^e^	–		–	N/A
C	300^f^	–		–	N/A

*AE* adverse event, *aGvHD* acute graft-versus-host disease, *MedDRA* Medical Dictionary for Regulatory Activities, *PT* preferred term, *TEAE* treatment-emergent adverse event.

^a^An event of death was reported as the AE as the cause of death was unknown.

^b^GvHD or complications of aGvHD.

^c^Death occurred 18 weeks (126 days) after the last dose of study drug.

Last assessment ^d^day 255. ^e^day 276. ^f^day 239.

## Discussion

The primary objective of this phase 2a study was to characterize the efficacy, tolerability, and safety of vedolizumab to identify a recommended dose for further evaluation of the treatment of SR intestinal aGvHD. While early clinical benefit (64.7% OR rate at day 15) was observed in approximately two-thirds of participants, less than half obtained an OR in intestinal aGvHD by day 28, with a significant number (*n* = 8) of participants dying from complications of aGvHD. An early review (prior to day 28) of patient-level data for the 17 enrolled participants concluded that the expected efficacy outcome for the study would not be achieved and the decision was made to terminate the study prior to completion of planned enrollment.

There are a number of potential reasons for the lack of a satisfactory OR in this study. Most notably, all participants had SR aGvHD with lower intestinal involvement, with a strict definition of SR, and approximately 70% had severe GI (Stage 3: 47.1%; Stage 4: 23.5%). Moreover, more than 70% of participants had SR Grade C or D aGvHD, and ~30% had multiorgan involvement at baseline. Among patients with aGvHD intestinal tract involvement, GI Stage 3–4 and SR aGvHD in particular are associated with substantial morbidity and mortality, with a significantly increased risk of infections, and a limited response to salvage therapies [5, 34–38]. The significant complications inherent in patients with such disease, including early death and premature discontinuation of study drug, can possibly impair the ability to evaluate efficacy compared with other SR aGvHD studies, which have included patients without intestinal involvement.

Given the data from this study, it is also certainly possible that treatment with vedolizumab was unable to exert an effect rapid enough and/or of significant magnitude to allow the injured GI epithelium to recover sufficiently and prevent disease-related complications. It has been proposed that if enough tissue injury has occurred, T-cell migration to the GI endothelium is of limited importance, and α4β7 integrin is no longer needed for the propagation of aGvHD [39]. From this perspective, vedolizumab might not be expected to exert a significant therapeutic effect in advanced disease. This hypothesis is supported by a recent study by Fu et al., which highlighted the key role played by the α4β7-MAdCAM-1 interaction in the early recruitment of donor T cells to the intestinal stem cell compartment [40]. Therefore, vedolizumab may have greater therapeutic benefit if administered as initial or preventative therapy.

A number of case series on the use of vedolizumab in SR intestinal aGvHD have reported variable outcomes [9, 26, 39, 41, 42]. Fløisand et al. initially reported a 100% OR rate in six patients with SR intestinal aGvHD treated with vedolizumab 300 mg, with two deaths (due to sepsis/acute respiratory distress syndrome and multiorgan failure) after a median follow-up time of 10 months [26]. Contrastingly, Bukauskas et al. reported five patients with SR aGvHD with intestinal involvement who were treated with vedolizumab and who died at a median of 32 days (range, 7–172 days) after starting therapy. All deaths were attributed to infectious complications with persistent aGvHD symptoms. Partial responses were observed in only two patients [41]. Similarly, Coltoff et al. reported high mortality rates in a retrospective case series of nine patients with SR intestinal aGvHD treated with vedolizumab 300 mg, with only one patient surviving beyond 60 days, with most patients dying of infection [42].

There have been two recent large retrospective multicenter case series published. Danylesko et al. described 29 patients with SR intestinal aGvHD treated with vedolizumab IV 300 mg and demonstrated OR rates of 79% (28% complete response, 52% partial response) after 7–10 days. Twelve patients (41%) were able to discontinue immunosuppressive therapy. Overall survival rates at 6 and 12 months were 41.4% and 27.5%, respectively, with the majority of successful outcomes observed in patients who were treated early in their disease course [39]. Fløisand et al. described a series of 29 patients with SR aGvHD treated with vedolizumab, reporting an OR rate of 64% (6–10 weeks after initiation of drug) and a 6-month overall survival rate of 54% [9].

Taken together, the early OR results observed in this study, along with the anecdotal evidence from retrospective case studies, justify pursuing the evaluation of vedolizumab for lower intestinal SR GvHD. However, considering the mechanism of action and kinetics of GI epithelial regeneration in the context of aGvHD, vedolizumab may be more effective if administered earlier in the disease course, such as for prevention, pre-emptive use, or initial therapy.

With regard to safety, observed adverse events were comparable with prior reports in a patient population with SR GvHD [43]. The mortality rate observed in this trial was comparable to historical series of patients with SR aGvHD with intestinal involvement [44, 45]. In the current study, no safety signals were identified that were associated with the use of either dose of vedolizumab, and the reported adverse events were not related to immunogenicity because all participants were negative for anti-vedolizumab antibodies.

Overall, this study was unable to demonstrate a positive proof-of-concept in efficacy in participants with SR GvHD treated with vedolizumab. While early and durable responses to intestinal aGvHD following treatment with vedolizumab were observed in some participants, vedolizumab was unable to generate a satisfactory response in this study. Evaluating the true efficacy of vedolizumab in patients with advanced severe intestinal aGvHD and associated complications is clearly challenging and perhaps not realistically possible. It may be that vedolizumab may be more suited to use for intestinal GvHD prevention, and this is currently being evaluated in a phase 3 clinical trial (NCT03657160).

The observations made in this study are nevertheless important, as they provide vital information on the severity of SR intestinal aGvHD and management of complications that need to be considered when designing and conducting future studies in such patients if positive efficacy outcomes are to be demonstrated.

## Supplementary information

Supplementary material

## Data Availability

Data pertaining to this study and the associated study protocol may be found at ClinicalTrials.gov (identifier: NCT02993783). Reuse of these data is not permitted. Additional data that support the findings of this study are available from Takeda Pharmaceuticals International upon reasonable request.
