# Supplementary material for: A phase 2a randomized clinical trial of intravenous vedolizumab for the treatment of steroid-refractory intestinal acute graft-versus-host disease
Source: Bone Marrow Transplant. 2021 Jun 9;56(10):2477–88. doi: 10.1038/s41409-021-01356-0 (PMC8486663; doi:10.1038/s41409-021-01356-0)
Supplement: Supplementary file 1 — Supplementary material [file 41409_2021_1356_MOESM1_ESM.docx]

# Supplementary material

A **phase 2a** randomized clinical trial **of intravenous vedolizumab for the treatment of steroid-refractory intestinal acute graft-versus-host disease**

Yngvar Fløisand, Mark A. Schroeder, Patrice Chevallier, Dominik Selleslag, Steven Devine, Anne S. Renteria, Mohamad Mohty, Ibrahim Yakoub-Agha, Chunlin Chen, Andrejus Parfionovas, Syed Quadri, Johan Jansson, Mona Akbari, Yi-Bin Chen

Supplementary Table 1 Definitions for assessing response in treatment of acute graft-versus-host disease [1].

| **Response** | **Skin** | **Liver** | **Gastrointestinal tract** |
| --- | --- | --- | --- |
| Complete response | No skin involvement | No hepatic involvement | No intestinal involvement |
| Very good partial response | No rash, or residual erythematous rash involving <25% of the body surface, without bullae (excluding residual faint erythema and hyperpigmentation) | Total serum bilirubin concentration <2 mg/dL or <25% of baseline at enrolment | • Subject tolerates food or enteral feeding  • Predominantly formed stools  • No overt gastrointestinal bleeding or abdominal cramping  • No more than occasional nausea or vomiting |
| Partial response | Improvement of one GvHD stage in one or more organs without progression in any organ | | |

*GvHD* acute graft-versus-host disease.

Supplementary Table 2 Detailed inclusion and exclusion criteria.

This table is available separately via the online version of this article.

| **Inclusion criteria** | **Exclusion criteria** |
| --- | --- |
| 1. Male or female participants aged 18 years or older. 2. Participants who had received one allogeneic hematopoietic cell transplantation, but not more than one allogeneic hematopoietic cell transplantation. 3. Participants with primary SR GvHD. SR disease was defined as worsening or no improvement in 5 to 7 days of treatment with methylprednisolone 2 mg/kg or equivalent or lack of a complete response after 14 days of primary treatment with methylprednisolone 2 mg/kg or equivalent. Note that participants who developed intestinal GvHD while receiving systemic therapy for other GvHD were still eligible after 5 to 7 days, even if the intestinal GvHD had not been present for the entire duration. Participants who may have received an increase in their steroid dose treatment (e.g., increased methylprednisolone from 1 mg/kg to 2 mg/kg) before enrollment were eligible, provided the participant met the definition of SR above. 4. ECOG Performance Status of 0 to 3. 5. Acute GvHD with intestinal disease involvement with a severity index of B, C, or D using the Blood and Marrow Transplant Clinical Trials Network-modified International Bone Marrow Transplant Registry Database index. Note that other organ involvement from aGvHD was also allowed. 6. Evidence of myeloid engraftment defined by absolute neutrophil count ≥0.5 × 10^9^/L on 3 consecutive days. 7. Creatinine clearance based on the Cockcroft–Gault estimate of ≥60 mL/min/1.73 m^2^ for participants with serum creatinine concentrations above institutional limits. 8. Sufficient cognitive ability to reliably complete the Risk Assessment and Minimization of progressive multifocal leukoencephalopathy questionnaire (i.e., PML checklist) at baseline.   Female participants who:   - were postmenopausal for at least 1 year before the screening visit, or - were surgically sterile, or - if they were of childbearing potential, agreed to practice one highly effective method of contraception and one additional effective (barrier) method at the same time, from the time of signing the informed consent through 18 weeks after the last dose of study drug, or - agreed to practice true abstinence, when this was in line with the preferred and usual lifestyle of the participant (periodic abstinence [e.g., calendar, ovulation, symptothermal, post ovulation methods], withdrawal, spermicides only, and lactational amenorrhea were not acceptable methods of contraception, Female and male condoms were not to be used together.)   Male participants, even if surgically sterilized (i.e., status post vasectomy), who:   - agreed to practice effective barrier contraception during the entire study treatment period and through 18 weeks after the last dose of study drug, or - agreed to practice true abstinence, when this was in line with the preferred and usual lifestyle of the participant. (Periodic abstinence [e.g., calendar, ovulation, symptothermal, post ovulation methods], withdrawal, spermicides only, and lactational amenorrhea were not acceptable methods of contraception. Female and male condoms were not to be used together.)   Voluntary written consent had to be given before performance of any study-related procedure not part of standard medical care, with the understanding that consent could be withdrawn by the participant at any time without prejudice to future medical care.  Suitable venous access for the study-required blood sampling, including PK and biomarker sampling. Participants with a planned central venous access device were allowed. | 1. Presence of chronic GvHD at screening (including acute-chronic overlap syndrome).   Relapsed disease after allogeneic hematopoietic cell transplantation.  Participants with hyperacute GvHD defined as onset of GvHD within the first 15 days following hematopoietic stem cell infusion.  Received systemic agents other than corticosteroids for treatment of aGvHD. GvHD prophylaxis agents (e.g., calcineurin inhibitors) may have been continued.  SR aGvHD beyond 28 days from primary treatment.  Participants with a positive PML subjective checklist had to be evaluated by a neurologist for possible PML before enrollment. Participants were to be excluded if PML could not be ruled out.  Evidence of encephalopathy at screening.  Evidence of severe hepatic veno-occlusive disease/sinusoidal obstruction syndrome.  Life expectancy of <3 weeks.  History of any major neurological disorders, including multiple sclerosis or neurodegenerative disease. Participants with a history of stroke or brain tumor within the past 3 years were also excluded.  Participants with active cytomegalovirus colitis  Participants that chronic HBV or HCV infection indicated by testing for positive HBV surface antigen, and/or HCV RNA.  Any identified congenital or acquired immunodeficiency (e.g., common variable immunodeficiency, human immunodeficiency virus infection, organ transplantation).  Positive *Clostridium difficile* toxin test on a stool sample or evidence of other intestinal pathogens (e.g., adenovirus) during screening.  Evidence of uncontrolled active systemic infection.   1. Any serious medical or psychiatric condition that, in the investigator’s or medical monitor’s opinion, could have potentially interfered with the completion of treatment according to the protocol.   Any unstable or uncontrolled cardiovascular, pulmonary, hepatic, renal, gastrointestinal (GI), genitourinary, hematological, coagulation, immunological, endocrine/metabolic, neurologic, or other medical disorder that, in the opinion of the investigator or medical monitor, would have confounded the study results or compromised participant safety.  History of hypersensitivity or allergies to vedolizumab or its components.  If female, the participant was pregnant or lactating or intended to become pregnant before, during, or within 18 weeks after participating in this study; or intended to donate ova during such time period.  If male, the participant intended to donate sperm during this study or for 18 weeks thereafter. |

*ECOG* Eastern Cooperative Oncology Group, *aGvHD* acute graft-versus-host disease, *GvHD* graft-versus-host disease, *HBV* hepatitis B, *HCV* hepatitis C, *PK* pharmacokinetics, *PML* progressive multifocal leukoencephalopathy, *RNA,* ribonucleic acid, *SR* steroid-refractory.

Supplementary Table 3 Clinical staging and grading of graft-versus-host disease.

| **Clinical stages of aGvHD [2]** | | | | | |
| --- | --- | --- | --- | --- | --- |
| **Stage** | **Skin** |  | **Liver**  **Bilirubin: SI units** |  | **Gastrointestinal tract**  **Diarrhea/day** |
| 1 | Maculopapular rash  <25% of body surface |  | 34–50 μmol/L  (2–3 mg/dL) |  | >500 mL |
| 2 | Maculopapular rash  25–50% of body surface |  | 51–102 μmol/L  (3.1–6 mg/dL) |  | >1000 mL |
| 3 | Rash >50% of body surface |  | 103–225 μmol/L  (6.1–15 mg/dL) |  | >1500 mL |
| 4 | Generalized erythroderma with bullous formation |  | >255 μmol/L  (>15 mg/dL) |  | Severe abdominal pain, with or without ileus |
| **Criteria for IBMTR Severity Index for aGvHD [3]** | | | | | |
| **Grade** | **Skin stage (max)** |  | **Liver stage (max)** |  | **Gastrointestinal stage (max)** |
| A | 1 |  | 0 |  | 0 |
| B | 2 | or | 1–2 | or | 1–2 |
| C | 3 | or | 3 | or | 3 |
| D | 4 | or | 4 | or | 4 |

*aGvHD* acute graft-versus-host disease, *IBMTR* International Bone Marrow Transplant Registry database.

Acute GvHD using the Blood and Marrow Transplant Clinical Trials Network-modified IBMTR index for staging and grading aGvHD was assessed at screening and on Days 1, 7, 15, 22, 28, 36, 43, 71, and 99, at 4-, 5-, 6-, and 9-month follow-up visits, and at the 12-month/end of study visit. Assessment of aGvHD was also performed during any extension dose or unscheduled visits should these occur.

Supplementary Table 4 PK sample collection.

| **Time of collection** | **Day of blood sample collection** | | | | | | | | | | | | | | | | | | | |
| --- | --- | --- | --- | --- | --- | --- | --- | --- | --- | --- | --- | --- | --- | --- | --- | --- | --- | --- | --- | --- |
|  | 1 | 2 | 3 | 5 | 7 | 9 | 11 | 15 | 16 | 18 | 20 | 22 | 24 | 28 | 32 | 36 | 40 | 43 | 71 | 99 |
| Non-dosing days^a^ |  |  | × | × | × | × | × |  |  | × | × | × | × | × | × | × | × |  |  |  |
| Predose | × |  |  |  |  |  |  | × |  |  |  |  |  |  |  |  |  | × | × | × |
| 30 min post dose (± 5 min) | × |  |  |  |  |  |  |  |  |  |  |  |  |  |  |  |  | × | × | × |
| 1 h post dose (±10 min) |  |  |  |  |  |  |  |  |  |  |  |  |  |  |  |  |  | × | × | × |
| 2 h post dose (±20 min) | × |  |  |  |  |  |  | × |  |  |  |  |  |  |  |  |  | × | × | × |
| 12 h post dose (±30 min) | × |  |  |  |  |  |  | × |  |  |  |  |  |  |  |  |  |  |  |  |
| 24 h post dose (±60 min) |  | × |  |  |  |  |  |  | × |  |  |  |  |  |  |  |  |  |  |  |

*PK* pharmacokinetic.

^a^Once a participant had completed the inpatient period (as determined by the investigator), sample collection may have been aligned with clinic visits. Blood samples (one 5 mL sample per scheduled time) for PK analysis should not have been collected from the arm where the vedolizumab infusion was administered. All PK samples should have been collected within 10% of nominal time; however, samples collected outside this margin were not considered protocol deviations.

Supplementary Table 5. Characteristics of participants (A), donors (B), and graft-versus-host disease (C).

This table is available separately via the online version of this article.

| **Treatment group** | **Vedolizumab 300 mg** | | | | | | | | **Vedolizumab 600 mg** | | | | | | | | |
| --- | --- | --- | --- | --- | --- | --- | --- | --- | --- | --- | --- | --- | --- | --- | --- | --- | --- |
| **Participant** | **A** | **B** | **C** | **D** | **E** | **F** | **G** | **H** | **I** | **J** | **K** | **L** | **M** | **N** | **O** | **P** | **Q** |
| **A – Participant characteristic** |  |  |  |  |  |  |  |  |  |  |  |  |  |  |  |  |  |
| **Gender** |  |  |  |  |  |  |  |  |  |  |  |  |  |  |  |  |  |
| Male | × |  |  |  |  |  | × |  |  | × |  |  | × | × |  | × | × |
| Female |  | × | × | × | × | × |  | × | × |  | × | × |  |  | × |  |  |
| **Participant age (years)** | 47 | 59 | 56 | 55 | 45 | 40 | 57 | 74 | 70 | 34 | 62 | 52 | 66 | 57 | 61 | 61 | 68 |
| **Underlying disease** |  |  |  |  |  |  |  |  |  |  |  |  |  |  |  |  |  |
| AML or related precursor neoplasm |  |  | × | × |  | × |  |  |  |  |  |  |  | × |  |  |  |
| Mature B-cell neoplasm |  |  |  |  |  |  |  |  | × |  |  |  |  |  |  |  |  |
| Myelodysplastic or myeloproliferative neoplasm |  |  |  |  |  |  |  |  |  |  | × |  |  |  |  |  |  |
| Myelodysplastic syndrome |  | × |  |  |  |  |  | × |  |  |  |  |  |  | × |  | x |
| Precursor lymphoid neoplasm |  |  |  |  |  |  |  |  |  |  |  |  |  |  |  | × |  |
| Other^a^ | i |  |  |  | ii |  | iii |  |  | iv |  | v | vi |  |  |  |  |
| **Conditioning regimen** |  |  |  |  |  |  |  |  |  |  |  |  |  |  |  |  |  |
| Myeloablative transplant | × |  | × |  |  | × |  | × |  |  |  |  | × | × |  |  | × |
| Non-myeloablative or reduced-intensity transplant |  | × |  | × | × |  | × |  | × | × | × | × |  |  | × | × |  |

| **B – Donor characteristic** |  |  |  |  |  |  |  |  |  |  |  |  |  |  |  |  |  |
| --- | --- | --- | --- | --- | --- | --- | --- | --- | --- | --- | --- | --- | --- | --- | --- | --- | --- |
| **Relationship to participant** |  |  |  |  |  |  |  |  |  |  |  |  |  |  |  |  |  |
| Biological | × | × |  |  |  |  |  | × |  | × |  | × |  |  |  |  |  |
| Non-biological |  |  | × | × | × | × | × |  | × |  | × |  | × | × | × | × | × |
| **HLA compatibility** |  |  |  |  |  |  |  |  |  |  |  |  |  |  |  |  |  |
| Matched | × |  | × |  | × | × | × | × | × |  |  |  | × | × | × | × | × |
| Mismatched |  |  |  | × |  |  |  |  |  |  | × |  |  |  |  |  |  |
| Haploidentical |  | × |  |  |  |  |  |  |  | × |  | × |  |  |  |  |  |
| **Source of stem cells** |  |  |  |  |  |  |  |  |  |  |  |  |  |  |  |  |  |
| Bone marrow |  |  |  |  |  | × |  |  |  |  |  |  |  | × |  |  |  |
| Peripheral blood | × | × | × | × | × |  | × | × | × | × | × | × | × |  | × | × | × |
| **C – GvHD characteristics** |  |  |  |  |  |  |  |  |  |  |  |  |  |  |  |  |  |
| **Involvements** |  |  |  |  |  |  |  |  |  |  |  |  |  |  |  |  |  |
| Intestinal only | × | × | × | × | × | × |  | × |  | × |  | × |  | × | × |  | × |
| Skin and intestinal |  |  |  |  |  |  | × |  | × |  | × |  |  |  |  |  |  |
| Liver and intestinal |  |  |  |  |  |  |  |  |  |  |  |  | × |  |  |  |  |
| Skin, liver, and intestinal |  |  |  |  |  |  |  |  |  |  |  |  |  |  |  | × |  |
| **Gastrointestinal tract^b^** |  |  |  |  |  |  |  |  |  |  |  |  |  |  |  |  |  |
| Stage 1 |  |  |  | × |  |  |  |  |  | × | × |  | × |  |  |  |  |
| Stage 2 |  |  | × |  |  |  |  |  |  |  |  |  |  |  |  |  |  |
| Stage 3 | × | × |  |  |  |  | × | × |  |  |  |  |  | × | × | × | × |
| Stage 4 |  |  |  |  | × | × |  |  | × |  |  | × |  |  |  |  |  |
| **Grade^c^** |  |  |  |  |  |  |  |  |  |  |  |  |  |  |  |  |  |
| Grade A |  |  |  |  |  |  |  |  |  |  |  |  |  |  |  |  |  |
| Grade B |  |  | × | × |  |  |  |  |  | × | × |  | × |  |  |  |  |
| Grade C | × | × |  |  |  |  | × | × |  |  |  |  |  | × | × | × | × |
| Grade D |  |  |  |  | × | × |  |  | × |  |  | × |  |  |  |  |  |
| **CMV IgG antibody match, participant/donor** |  |  |  |  |  |  |  |  |  |  |  |  |  |  |  |  |  |
| Positive/positive |  |  | × |  |  |  | × |  |  |  |  |  |  |  | × |  |  |
| Positive/negative |  |  |  |  | × |  |  | × | × |  |  |  |  |  |  |  | × |
| Negative/positive | × |  |  | × |  |  |  |  |  | × |  | × |  |  |  |  |  |
| Negative/negative |  | × |  |  |  | × |  |  |  |  | × |  | × | × |  | × |  |

*AML* acute myeloid leukemia, *CMV* cytomegalovirus, *GI* gastrointestinal, GvHD graft-versus-host disease, IgG immunoglobulin G.

^a^*i* granulocytic sarcoma, *ii* systemic mastocytosis, *iii* mantle cell lymphoma, i*v* Hodgkin disease, *v* chronic myelomonocytic leukemia, *vi* myelofibrosis.

^b^Stage is based on aGvHD clinical stage criteria which consider the extent and severity of involvement for each organ, measured at the screening visit. GI staging: *0* no GI tract involvement, *1* >500 mL diarrhea/day, *2* >1000 mL diarrhea/day, *3* >1500 mL diarrhea/day, *4* severe abdominal pain with or without ileus. Stages of involvement for different organs are then combined to generate an overall clinical grade.

^c^Grades were derived using the Blood and Marrow Transplant Clinical Trials Network-modified International Blood and Marrow Transplant Research database index.

Supplementary Table 6 Most commonly reported (≥10% of all participants) Grade 3 or higher treatment-emergent adverse events.

This table is available separately via the online version of this article.

|  | Vedolizumab 300 mg (n = 8), n (%) | Vedolizumab 600 mg (n = 9), n (%) | Total  (N = 17), n (%) |
| --- | --- | --- | --- |
| SOC and Preferred Term |  |  |  |
| Blood and lymphatic system disorders | 4 (50) | 5 (55.6) | 9 (52.9) |
| Anemia | 3 (37.5) | 3 (33.3) | 6 (35.3) |
| Neutropenia | 0 | 3 (33.3) | 3 (17.6) |
| Thrombocytopenia | 1 (12.5) | 1 (11.1) | 2 (11.8) |
| Gastrointestinal disorders | 3 (37.5) | 3 (33.3) | 6 (35.3) |
| Diarrhea | 1 (12.5) | 1 (11.1) | 2 (11.8) |
| Lower gastrointestinal hemorrhage | 1 (12.5) | 1 (11.1) | 2 (11.8) |
| General disorders and administration site conditions | 2 (25.0) | 2 (22.2) | 4 (23.5) |
| Immune system disorders | 1 (12.5) | 5 (55.6) | 6 (35.3) |
| Graft-versus-host disease | 0 | 3 (33.3) | 3 (17.6) |
| Infections and infestations | 6 (75.0) | 7 (77.8) | 13 (76.5) |
| Sepsis | 2 (25.0) | 1 (11.1) | 3 (17.6) |
| Cytomegalovirus colitis | 1 (12.5) | 1 (11.1) | 2 (11.8) |
| Enterococcal sepsis | 1 (12.5) | 1 (11.1) | 2 (11.8) |
| *Escherichia* infection^a^ | 0 | 2 (22.2) | 2 (11.8) |

| Investigations | 3 (37.5) | 5 (55.6) | 8 (47.1) |
| --- | --- | --- | --- |
| Blood bilirubin increased | 2 (25.0) | 1 (11.1) | 3 (17.6) |
| Platelet count decreased | 2 (25.0) | 1 (11.1) | 3 (17.6) |
| White blood cell count decreased | 1 (12.5) | 2 (22.2) | 3 (17.6) |
| Metabolism and nutrition disorders | 4 (50.0) | 3 (33.3) | 7 (41.2) |
| Hypokalemia | 1 (12.5) | 2 (22.2) | 3 (17.6) |
| Hypoalbuminemia | 2 (25.0) | 1 (11.1) | 3 (17.6) |
| Hyperglycemia | 2 (25.0) | 0 | 2 (11.8) |
| Neoplasms benign, malignant, and unspecified (incl cysts and polyps) | 1 (12.5) | 1 (11.1) | 2 (11.8) |
| Nervous system disorders | 0 | 2 (22.2) | 2 (11.8) |
| Renal and urinary disorders | 2 (25.0) | 1 (11.1) | 3 (17.6) |
| Acute kidney injury | 1 (12.5) | 1 (11.1) | 2 (11.8) |
| Respiratory, thoracic, and mediastinal disorders | 3 (37.5) | 3 (33.3) | 6 (35.3) |
| Hypoxia | 1 (12.5) | 1 (11.1) | 2 (11.8) |
| Respiratory failure | 2 (25.0) | 0 | 2 (11.8) |
| Skin and subcutaneous tissue disorders | 1 (12.5) | 1 (11.1) | 2 (11.8) |
| Vascular disorders | 1 (12.5) | 5 (55.6) | 6 (35.3) |
| Hypotension | 1 (12.5) | 3 (33.3) | 4 (23.5) |
| Hematoma | 0 | 2 (22.2) | 2 (11.8) |
| TEAE |  |  |  |
| Grade 3 or higher | 8 (100.0) | 9 (100.0) | 17 (100.0) |
| Related | 0 | 1 (11.1) | 1 (5.9) |
| Not related | 8 (100.0) | 8 (88.9) | 16 (94.1) |
| Leading to study drug discontinuation | 2 (25.0) | 2 (22.2) | 4 (23.5) |
| Serious TEAE |  |  |  |
| Grade 3 or higher | 6 (75.0) | 9 (100.0) | 15 (88.2) |
| Related | 0 | 1 (11.1) | 1 (5.9) |
| Not related | 6 (75.0) | 8 (88.9) | 14 (82.4) |
| Leading to study drug discontinuation | 1 (12.5) | 1 (11.1) | 2 (11.8) |
| AEs leading to death^b^ | 4 (50.0) | 9 (100.0) | 13 (76.5) |

Data presented are from the safety analysis set.

A TEAE is defined as any AE that starts or worsens on or after administration of the first dose of study drug, and no more than 18 weeks (126 days) after the last dose of study drug. Percentages are based on the number of participants in the safety analysis set. Participants with ≥1 TEAE within a level of MedDRA term are counted only once in that level. Severity (toxicity grade) for each AE was determined using the NCI CTCAE, version 4.03, effective date 14 June 2010.

*AE* adverse event, *MedDRA* Medical Dictionary for Regulatory Activities, *NCI CTCAE* National Cancer Institute Common Terminology Criteria for Adverse Events, *TEAE* treatment-emergent adverse event.

^a^One participant in the 600 mg group with an event of *Escherichia* infection, sepsis was initially reported as related to *E. coli* infection but was later attributed to a later event of *Citrobacter* infection.

^b^An additional participant in the 300 mg group died after the end of the treatment-emergent period (i.e., 18 weeks [126 days] after the last dose of study drug).

Supplementary Table 7 Most commonly reported (≥10% of all participants) treatment-emergent adverse events of special interest: infections.

This table is available separately via the online version of this article.

|  | Vedolizumab 300 mg (n = 8), n (%) | Vedolizumab 600 mg (n = 9), n (%) | Total  (N = 17), n (%) |
| --- | --- | --- | --- |
| Preferred Term |  |  |  |
| Participants with any infection AESI | 7 (87.5) | 8 (88.9) | 15 (88.2) |
| Cytomegalovirus infection | 2 (25.0) | 1 (11.1) | 3 (17.6) |
| Sepsis | 2 (25.0) | 1 (11.1) | 3 (17.6) |
| BK virus infection | 1 (12.5) | 1 (11.1) | 2 (11.8) |
| Cytomegalovirus colitis | 1 (12.5) | 1 (11.1) | 2 (11.8) |
| Enterococcal infection | 1 (12.5) | 1 (11.1) | 2 (11.8) |
| Enterococcal sepsis | 1 (12.5) | 1 (11.1) | 2 (11.8) |
| *Escherichia* infection | 0 | 2 (22.2) | 2 (11.8) |
| *Klebsiella* infection | 1 (12.5) | 1 (11.1) | 2 (11.8) |
| Staphylococcal infection | 0 | 2 (22.2) | 2 (11.8) |
| Urinary tract infection enterococcal | 1 (12.5) | 1 (11.1) | 2 (11.8) |
| Participants with any serious infection AESI | 5 (62.5) | 4 (44.4) | 9 (52.9) |
| Sepsis | 2 (25.0) | 1 (11.1) | 3 (17.6) |
| Enterococcal sepsis | 1 (12.5) | 1 (11.1) | 2 (11.8) |
| Participants with any Grade 3 or higher infection AESI | 6 (75.0) | 7 (77.8) | 13 (76.5) |
| Cytomegalovirus colitis | 1 (12.5) | 1 (11.1) | 2 (11.8) |
| Enterococcal sepsis | 1 (12.5) | 1 (11.1) | 2 (11.8) |
| *Escherichia* infection | 0 | 2 (22.2) | 2 (11.8) |
| Sepsis | 2 (25.0) | 1 (11.1) | 3 (17.6) |

Data presented are from the safety analysis set.

A TEAE is defined as any AE that starts or worsens on or after administration of the first dose of study drug, and no more than 18 weeks (126 days) after the last dose of study drug. Percentages are based on the number of participants in the safety analysis set. Participants with ≥1 AE within a level of MedDRA term are counted only once in that level. Severity (toxicity grade) for each AE was determined using the NCI CTCAE, version 4.03, effective date 14 June 2010.

*AE,* adverse event, *AESI* adverse event of special interest, *MedDRA* Medical Dictionary for Regulatory Activities, *NCI CTCAE* National Cancer Institute Common Terminology Criteria for Adverse Events, *PT* Preferred Term, *SOC* System Organ Class, *TEAE* treatment-emergent adverse event.

## References

1. Martin PJ, Bachier CR, Klingemann HG, McCarthy PL, Szabolcs P, Uberti JP et al. Endpoints for clinical trials testing treatment of acute graft-versus-host disease: a joint statement. *Biol Blood Marrow Transplant* 2009;15:777-784

2. Przepiorka D, Weisdorf D, Martin P, Klingemann HG, Beatty P, Hows J et al. 1994 consensus conference on acute GvHD grading. *Bone Marrow Transplant.* 1995;15:825-828

3. Rowlings PA, Przepiorka D, Klein JP, Gale RP, Passweg JR, Henslee-Downey PJ et al. IBMTR Severity Index for grading acute graft-versus-host disease: retrospective comparison with Glucksberg grade. *Br J Haematol* 1997;97:855-864
